# Glaucoma detection training of community healthcare workers using portable devices in Nigeria

**DOI:** 10.3389/fpubh.2025.1740419

**Published:** 2026-01-09

**Authors:** Farouk Garba, Winifred Nolan, Matthew J. Burton, Victor H. Hu, Ajefu Rose Ada, Kenneth Ezurike, Micheal Ochefu Ajefu, Fatima Kyari

**Affiliations:** 1 International Centre for Eye Health, London School of Hygiene and Tropical Medicine, London, United Kingdom; 2 Department of Ophthalmology, Faculty of Clinical Sciences, College of Medical Sciences, Ahmadu Bello University, Zaira, Nigeria; 3 NIHR Moorfields Biomedical Research Centre, London, United Kingdom; 4 College of Health Sciences, University of Abuja, Abuja, Nigeria; 5 Institute for Medical Research and Training, University of Abuja, Abuja, Nigeria

**Keywords:** community health workers, detection, glaucoma, Nigeria, training

## Abstract

**Background:**

Glaucoma, an irreversible blinding eye disease caused by optic nerve damage, remains a major public health challenge in sub-Saharan Africa, where it occurs earlier and progresses more aggressively than in other regions. Accessibility and affordability remain major barriers to timely diagnosis and treatment in low- and middle-income countries (LMICs).

**Purpose:**

To assess the ability of community health workers (CHWs) without prior ophthalmic training to use portable diagnostic tools for early detection and referral of possible glaucoma cases in Abuja, Nigeria.

**Methods:**

This feasibility study was conducted from June to September 2024 in three community health centres in Abuja. Fifteen CHWs (14 females, 1 male) were trained over 3 days using didactic lectures, practical demonstrations, and hands-on sessions on four portable devices: PEEK Acuity, iCare tonometer, Eyecatcher visual field analyser, and Remidio handheld fundus camera. Pre- and post-training assessments were conducted, with an 80% pass mark required. A simplified scoring system based on test findings guided referral decisions.

**Results:**

All CHWs achieved scores of 80% or higher post-training, showing significant improvement in glaucoma knowledge and testing proficiency. Initial challenges with the iCare tonometer and fundus camera improved with practice.

**Conclusion:**

Community health workers can effectively use portable diagnostic tools for glaucoma screening and referral in primary care settings. Their involvement could enhance early detection and contribute substantially to reducing glaucoma-related blindness in resource-limited settings.

## Introduction

Primary care refers to clinical services delivered to people in the community by trained health professionals which could range from community or family physicians, community nurses, Community Health-care Workers (CHWs) and others (1, 2). In high-income countries, primary care set up is more catered for with family physicians providing care at this level of health care. However, in Low and Middle Income Countries (LMICs), nurses or community health care workers are mostly the first line in the Primary Healthcare Centres (PHCs) (2).

In 2019, the World Report on Vision (WRV) by the World Health Organization (WHO) called on countries to place renewed emphasis on integrating Primary Eye Care (PEC) services into primary healthcare (3). This recommendation aimed to address barriers to accessibility and affordability that hinder the delivery of basic eye health services in low- and middle-income countries (particularly in sub-Saharan Africa and South Asia) (3). The WHO Global Strategy on Human Resources for Health: Workforce 2030, adopted by the World Health Assembly in 2016, encourages countries to adopt a diverse, sustainable skills mix, harnessing the potential of CHWs in interprofessional primary care teams (4). The training of CHWs is geared towards service delivery targeted mostly towards preventive medicine interlaced with basic knowledge of maternal and child health services ranging from antenatal to immunisation services (1, 2, 4). They are also trained to attend to some uncomplicated endemic illnesses malaria and diarrhoeal diseases (1–4).

Glaucoma is the second leading cause of blindness (second to cataract) and the number one cause of irreversible blindness (5–7). Global prevalence studies have reported 64.3 million individuals were diagnosed with glaucoma worldwide in 2013 and it is projected to rise to 73 million by 2020 (8). Primary Open Angle Glaucoma (POAG) is the commonest type, with close to 60 million people affected in 2016 and is expected to rise to 65.5 million by 2020 (8). The life expectancy is increasing worldwide and with the growing number of older adult population, it is projected that 111.8 million people will have glaucoma in 2040 (8). Currently available treatments cannot reverse glaucomatous damage to the visual system; however, early diagnosis and treatment can prevent progression of the disease. In LMICs, up to 35% of patients present to the hospital blind in at least one eye (2, 9). In study (10) conducted in Ghana found nearly 24% of patients were blind in at least one eye at first presentation. Another study (11) in Tanzania, also reported 44.7% of patients presented with severe VF loss (< −20 dB), compared to 4.6% in England.

This study aims to assess the ability of community healthcare workers, without prior specialised ophthalmic training, to use portable diagnostic tools, in combination with a simple scoring system, to identify adults with possible glaucoma in the community, and to refer them appropriately for further evaluation and care in Abuja, Nigeria.

### Objectives of the study

- To assess if the CHW can easily operate portable devices for the detection of glaucoma
- To assess the percentage of CHW that could pass post assessment training at first sitting
- To determine any difficulties with the use of the devices

## Methodology

### Study design

This was a observational descriptive design study conducted in 3 selected PHCs in Abuja Nigeria.

### Setting

Three PHCs in Abuja, Nigeria (Kuje, Gwagwalada and Garki) were selected through purposive and convenient sampling. Security concerns in the country restricted travel to remote areas, so these centres were chosen as the safest and most accessible locations for the study. CHWs were allocated by the Matron in-charge of the centre based on availability. None had prior experience working in eye care/ophthalmic training or with ophthalmic devices. A separated area/office was allocated for this project in each PHC. The training was delivered by a consultant ophthalmologist and two medical doctors experienced in the use of portable ophthalmic devices. It combined theoretical instruction with practical, hands-on sessions. The CHWs were trained to operate a range of portable eye diagnostic devices, including the PEEK Acuity app for assessing visual acuity, the iCare tonometer for measuring intraocular pressure (IOP), the Eyecatcher for visual field testing, and the Remidio handheld fundus camera for optic disc photography. Additional materials used during the training included pen torches, laptops, a projector, data collection forms for recording and scoring findings, research questionnaires, as well as writing and sanitary supplies. Adults receiving care at the selected primary healthcare centres, as well as community health students undertaking their field placements during the study period, were invited to participate as subjects for training purposes. All participants received information about the study objectives and procedures, and provided written informed consent prior to volunteering.

### Data management

Data management covered all stages of data handling for this study. Paper based exam sheets and structured interviews were used to collect data, and findings from device use were recorded on assessment forms during training. Quantitative data were entered into Microsoft Excel (version 2019) and analysed using descriptive statistics in Excel and Stata (version 18.1). Qualitative feedback was examined through thematic coding. All paper forms were stored securely and digital files were password protected with access limited to the principal researcher.

### Ethical considerations

Approval from the following Ethics committees was obtained:

- The National Health Research Ethics Committee, Nigeria.
- The ethics committee of the London School of Hygiene & Tropical Medicine, UK.
- University of Abuja Teaching Hospital (UATH) Health Research Ethics Committee.

The study followed all relevant local legislation and institutional requirements. All participants gave written informed consent before taking part.

### Training schedule

These included a total of 6 sessions, 2 sessions per day for 3 days.

**Figure 1:**
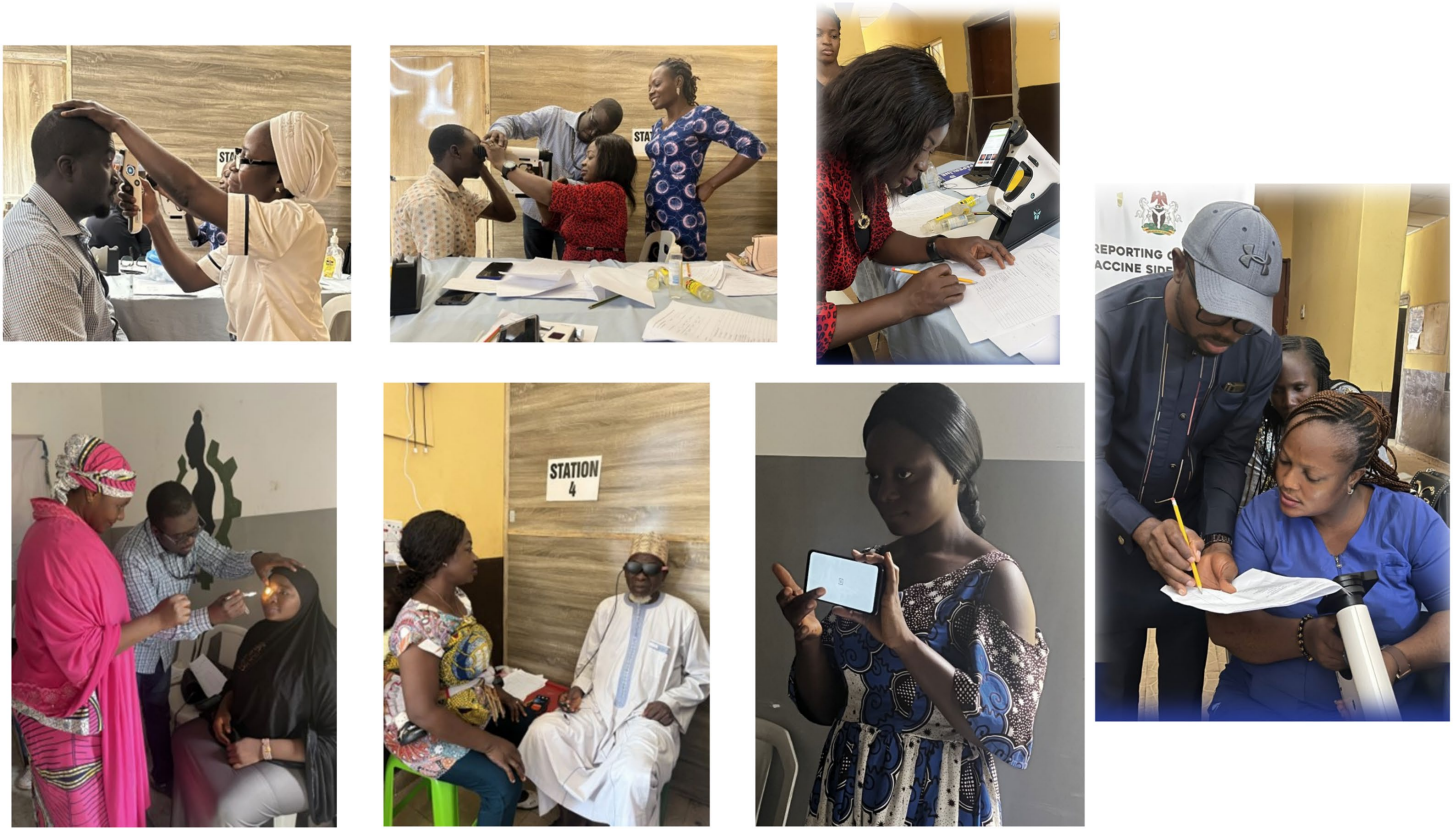
Pictures showing practical training of CHW.

The training program was organised over a three-day period, progressing from foundational knowledge acquisition to applied skill development as shown in Table 1 above. Day 1 focused on introductory activities, including participant introductions, a pretest, and didactic lectures on glaucoma and the objectives of the current project. This was followed by an introduction to portable diagnostic devices and the associated Standard Operating Protocols. Days 2 and 3 emphasized practical training, with structured, hands-on sessions that allowed participants to familiarise themselves with various tools such as PEEK, RAPD, iCare, Remidio, and Eyecatcher. Additionally, there was detailed instruction on the administration of research questionnaires and the logistical flow of patient stations. The training concluded with a post-test, practical evaluation using volunteer participants, and a feedback session. The inclusion of both formative (pretest) and summative (post-test) assessments, along with participant feedback, reflects a comprehensive approach to training evaluation. The design of the training facilitated a progressive and experiential learning environment, ensuring that participants developed both theoretical knowledge and practical competence (see Figure 1).

**Table 1:** Training timetable.

Day	Session	Topic
1	A	Introduction of members
		Pretest
		Lecture on glaucoma, presentation, investigations and management
		Question and answer
		Break
	B	Lecture on the current project
		Introducing the portable devices and
		Standard operating protocols
		Question and answer
2	C	Practical demonstration of how to use the portable devices
		How to complete research questionnaire
		Hands on training sessions I (PEEK, RAPD, iCare)
		Break
	D	Hand on training sessions II (Remidio and Eyecatcher)
		Description of flow of participants/stations
		Practical demonstration of flow of patients and how to fill questionnaire I
3	E	Practical demonstration of flow of patients and how to fill questionnaire II
		Practical demonstration of flow of patients and how to fill questionnaire III
		Practical demonstration of flow of patients and how to fill questionnaire IV
		Break
	F	Practical demonstration of flow of patients and how to fill questionnaire V
		Practical demonstration of flow of patients and how to fill questionnaire VI
		Post test
		End of training test using volunteer participants.
		Post training feedback form

### Questions and marking scheme for post-training questions

To evaluate knowledge acquisition following the training, a structured post-training test was administered to all participants. A detailed marking scheme was developed to ensure consistency, objectivity, and standardisation in scoring across all respondents. The test comprised seven questions, targeting both conceptual understanding and practical knowledge related to glaucoma and the use of portable diagnostic devices.

Each question was assigned a specific number of marks based on its complexity and the depth of response required. The marking criteria for each question were clearly delineated to minimise subjectivity and allow for reliable assessment.

A pass mark was set at 80%, corresponding to a minimum score of 16 out of 20. This threshold was determined to ensure that only participants who demonstrated a strong grasp of both theoretical and practical components of the training would proceed to field activities (see Table 2).

**Table 2:** Knowledge assessment questions, answers and marking scheme.

Question No.	Question	Correct answer(s)	Marking criteria	Total marks
**1**	What is glaucoma?	Glaucoma is an irreversible eye disease that causes optic nerve damage, leading to vision loss.	- Clear definition: 1 mark- Mention of optic nerve damage: 1 mark- Emphasis on irreversible vision loss: 1 mark	3
**2**	List four risk factors for glaucoma	Any 4 of:- Increased intraocular pressure (IOP)- Family history- Age (older adults)- African descent- Thin corneas- Eye injury/surgery- Long-term corticosteroid use	1 mark for each correct factor (up to 4)	4
**3**	List investigations needed to diagnose glaucoma	Any 3 of:- Measure IOP (e.g., iCare tonometer)- Fundus photography (e.g., Remidio)- Visual field test (e.g., Eyecatcher)- RAPD test- Gonioscopy (valid, not portable)	1 mark for each correct investigation (up to 3)	3
**4**	What are the treatment modalities for glaucoma?	Any 4 of:- Medications (eye drops)- Laser therapy (trabeculoplasty)- Surgery (trabeculectomy)- Lifestyle modifications (monitoring, avoiding steroids)	1 mark for each correct treatment (up to 4)	4
**5**	List available treatment(s) of glaucoma offered in a nearby tertiary/secondary centre	Any treatment available locally (e.g., medications, laser therapy, surgery)	Identifies one valid treatment: 2 marks	2
**6**	Can we use portable devices to detect, diagnose, and monitor glaucoma?	Yes	Correct answer: 1 mark	1
**7**	List three portable devices used for diagnosing glaucoma	- PEEK Acuity- iCare tonometer- Eyecatcher visual field analyzer- Remidio handheld fundus camera	1 mark for each correct device (up to 3)	3

### Practical assessment questions

The purpose of this practical assessment was to evaluate the competence of community health care workers in using portable devices for glaucoma detection, including the PEEK Acuity tester, iCare tonometer, Eyecatcher visual field analyser, and Remidio handheld fundus camera. Additionally, the assessment aimed to ensure that CHWs could appropriately interpret the results and make referrals based on their findings (see Table 3).

**Table 3:** Practical assessment questions and marking scheme.

Section	Task	Marking scheme	Maximum marks (50)
1. Device operation	Demonstrate correct use of: PEEK Acuity iCare tonometer Eyecatcher visual field analyser Remidio handheld fundus camera Pen torch for RAPD assessment	4 marks per task	20
2. Correctly assess and score	Visual acuity Intraocular pressure vCDR RAPD Perimetry	4 marks per task with exception of RAPD; 5 marks	21
3. Referral decision make appropriate referral decision based on total score of patients findings	Review in 12 months Review in 6 months Refer for further evaluation	1 marks per task	3
4. Device handling and cleaning	Explain and demonstrate correct cleaning and storage of: iCare Tonometer Eyecatcher Remidio handheld fundus camera	2 marks per task	6

The assessment was designed to test the CHWs on the following key areas:

- *Device Operation*: The CHWs were required to demonstrate their ability to operate the portable devices for glaucoma detection.
- *Interpretation of Results*: CHWs were tasked with scoring visual acuity, intraocular pressure, vertical cup-to-disc ratio, and perimetry results.
- *Referral Decision*: Based on the results, CHWs needed to make appropriate referral decisions.
- *Handling*: The assessment evaluated the CHWs’ knowledge on proper device handling and cleaning based on the standard operating protocol (SOP).

Competence was assessed using a 50-mark scheme across four domains. Device operation (20 marks), clinical assessment and scoring (21 marks), referral decision-making (3 marks), and device handling and cleaning (6 marks) were evaluated. This framework measured both technical proficiency and clinical judgement (see Table 4).

**Table 4:** Grading scheme for practical assessment.

Score range	Description
A (45–50)	The CHW demonstrates exceptional skills in operating diagnostic devices, scoring glaucoma risks accurately, and making timely and appropriate referrals. No further training is required for this area.
B (40–44)	The CHW is proficient with the devices and scoring system but should refine their handling of certain devices and practise making more confident referral decisions.
C (30–39)	The CHW performs adequately but requires additional practice, particularly in device operation and understanding when to refer patients for further evaluation. More hands-on training is recommended.
D (20–29)	The CHW shows limited competence and will need further training in device operation, scoring, and understanding referral guidelines. Consider additional support or follow-up training.
F (0–19)	The CHW is not proficient in operating the devices or interpreting results. Immediate remediation is necessary, and further hands-on training and support are strongly recommended.

This grading scheme helped in assessing the level of competency and identify areas where additional training may be needed to ensure effective glaucoma detection and referral in community health care settings.

### Feedback and recommendations

- *A (45–50)*: the CHW demonstrates exceptional skills in operating diagnostic devices, scoring glaucoma risks accurately, and making timely and appropriate referrals. No further training is required for this area.
- *B (40–44)*: the CHW is proficient with the devices and scoring system but should refine their handling of certain devices and practice making more confident referral decisions.
- *C (30–39)*: the CHW performs adequately but requires additional practice, particularly in device operation and understanding when to refer patients for further evaluation. More hands-on training is recommended.
- *D (20–29)*: the CHW shows limited competence and will need further training in device operation, scoring, and understanding referral guidelines. Consider additional support or follow-up training.
- *F (0–19)*: the CHW is not proficient in operating the devices or interpreting results. Immediate remediation is necessary, and further hands-on training and support are strongly recommended.

## Results

A total of 16 (15 females 1 male) CHWs were recruited and trained from 3 PHCs. Average age was 36 years (range; 22–52 years).

**Figure 2:**
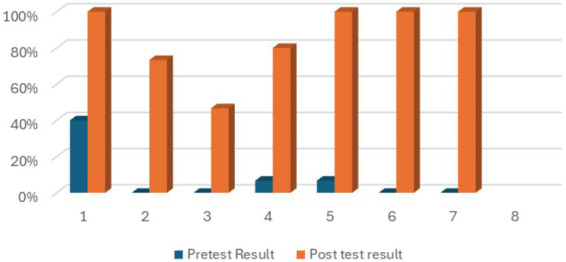
Bar chart showing pre and post test scores of CHW on knowledge of glaucoma.

Table 5, Figure 2 presents the pre- and post-test scores of community health workers (CHWs) on knowledge of glaucoma. Pretest scores ranged from 0 to 45% across the eight items. Item 1 recorded the highest pretest score (45%), while items 2, 3, 6, and 7 had the lowest (≤5%). Post-test scores were higher across all items, ranging from 50 to 100%. Items 1, 5, 6, 7, and 8 recorded 100% scores, while items 2, 3, and 4 recorded 75, 50, and 80%, respectively (see Table 6).

**Table 5:** Test questions showing knowledge of glaucoma before and after training among CHWs.

Test questions	Pretest result	Post test results
What is glaucoma?	40% (*n* = 6)	100% (*n* = 15)
List 4 risk factors for glaucoma	0	73.3% (*n* = 11)
List investigations needed to make a diagnosis of glaucoma	0	46.7% (*n* = 7)
What are the treatment modalities of glaucoma?	6.7% (*n* = 1)	80% (*n* = 12)
List available treatment(s) of glaucoma offered in a tertiary or secondary centre close to you.	6.7% (*n* = 1)	100% (*n* = 15)
Can we use portable devices to detect/diagnose and monitor glaucoma?	0	100% (*n* = 15)
If your answer is *yes* above? List 3 of these portable devices	0	100% (*n* = 15)

**Table 6:** Community health care workers practical training results and feedbacks.

S/N	No.	Age	Sex	Score	Major feedback from trainees
1.	CHW-1	50 years	F	82%	Difficulty in operating Remidio handheld fundus camera
2.	CHW-2	52 years	F	84%	Difficulty in operating Remidio handheld fundus camera and iCare tonometer
3.	CHW-3	44 years	F	92%	Enjoyed the training
4.	CHW-4	38 years	F	90%	Enlightening experience
5.	CHW-5	36 years	F	94%	Happy to participate
6.	CHW-6	42 years	F	92%	Good training, had difficulty focusing with Remidio handheld fundus camera
7.	CHW-7	48 years	F	96%	Excellent training
8.	CHW-8	46 years	F	80%	Difficulty in operating Remidio handheld fundus camera and iCare tonometer
9.	CHW-9	33 years	F	90%	Good
10.	CHW-10	24 years	F	92%	Excellent
11.	CHW-11	22 years	F	86%	Excellent
12.	CHW-12	26 years	F	98%	Learned a lot during the exercise
13.	CHW-13	28 years	F	90%	Good training
14.	CHW-14	25 years	M	86%	Challenges focusing disc with Remidio handheld fundus camera
15.	CHW-15	27 years	F	84%	Good training

## Discussion

This feasibility study explored the ability of CHWs without prior ophthalmic training to detect possible glaucoma using portable diagnostic devices in primary health care settings in Abuja, Nigeria. The findings demonstrated that CHWs could be trained within a short period to acquire both theoretical knowledge of glaucoma and practical competence in using devices such as the PEEK Acuity, iCare tonometer, Eyecatcher, and Remidio handheld fundus camera. The outcomes of this study provide important insights into the role of CHWs in expanding access to primary eye care and the potential of portable devices in facilitating early glaucoma detection in LMICs.

Baseline knowledge of glaucoma among CHWs was low, reflecting the inadequacy of eye health training in their routine curriculum. At pretest, only 40% of participants could define glaucoma correctly, and almost none could identify its risk factors, diagnostic investigations, or treatment options. These gaps in knowledge are consistent with previous reports from sub-Saharan Africa, where eye health is rarely integrated into community health worker training programmes, and awareness of glaucoma remains limited at both provider and community levels (8). The lack of awareness is concerning given the aggressive course of glaucoma in African populations and its contribution to irreversible blindness. Following training, knowledge improved substantially. All participants (100%) were able to define glaucoma correctly and to list the portable devices available for its detection. Furthermore, the proportion of CHWs able to identify risk factors, diagnostic investigations, and treatment modalities rose significantly, reflecting the effectiveness of the training intervention.

The training approach in this study, which combined lectures, demonstrations, and extensive hands-on sessions, proved particularly effective. Practical learning was essential, as CHWs are often more responsive to experiential methods than didactic lectures. By embedding opportunities for repeated practice, peer learning, and interactive demonstrations, the training created an environment where theoretical knowledge and technical skill developed in parallel. The use of pre- and post-tests, coupled with a structured practical evaluation, ensured that both cognitive understanding and operational competence were systematically assessed. This approach mirrors competency-based training frameworks advocated by WHO for strengthening CHW capacity in primary health care delivery (2).

While CHWs reported that the portable devices were generally easy to operate, difficulties were noted with specific tools, particularly the Remidio handheld fundus camera and, to a lesser extent, the iCare tonometer. Reported challenges included fatigue from prolonged use of the handheld fundus camera, difficulty focusing on the optic disc, and problems stabilising the tonometer during IOP measurement. These operational barriers highlight the importance of ergonomic considerations in device design, as well as the need for sufficient practice time during training to build user confidence and skill. Continuous training, ergonomic adjustments such as stabilisation stands, and refresher sessions could help mitigate these difficulties and enhance long-term usability.

Another challenge observed during training was the difficulty of detecting relative afferent pupillary defects (RAPD) in individuals with dark irises. Subtle pupillary responses are harder to detect in such cases, even for trained ophthalmologists. The use of high-quality instructional videos and repeated live demonstrations improved CHWs’ ability to recognise RAPD, but the limitation underscores the potential value of device-based solutions, such as infrared pupillometry, which can provide objective and quantitative assessments. Incorporating such technologies into portable diagnostic kits could further improve the accuracy and reliability of glaucoma detection in community settings.

The integration of glaucoma detection into primary care through CHWs has far-reaching implications for public health in sub-Saharan Africa. Glaucoma often presents late in this region, with many patients already blind in at least one eye at presentation. Studies in Ghana and Tanzania have shown that 24–45% of patients present with severe visual field loss at first consultation, compared to less than 5% in high-income countries such as England. This late presentation severely limits treatment options and increases the burden of irreversible blindness on individuals, families, and health systems.

Early identification and referral at the community level could therefore help reduce the proportion of late-stage glaucoma cases. By embedding glaucoma screening into routine primary care activities, CHWs could serve as the first line of defence against preventable blindness. This approach aligns with the WRV (2019), which emphasised the integration of primary eye care into primary health care systems, particularly in LMICs (3).

Portable devices are particularly well-suited to LMIC contexts due to their affordability, portability, and minimal maintenance requirements. Unlike traditional diagnostic tools that require specialised training, electricity, and hospital-based infrastructure, portable devices such as the iCare tonometer and Remidio handheld fundus camera can be used in rural or resource-limited settings. By equipping CHWs with these tools, eye health services can be decentralised and embedded into existing primary care systems without requiring large-scale infrastructural investments. This decentralisation is critical in Nigeria and other LMICs, where ophthalmologists are few and unevenly distributed, often concentrated in urban tertiary hospitals.

The use of CHWs in glaucoma detection is also consistent with the WHO Global Strategy on Human Resources for Health: Workforce 2030, which advocates for optimising the potential of CHWs within multidisciplinary primary care teams. By expanding their roles beyond maternal and child health, malaria, and infectious disease control, CHWs can contribute to the management of non-communicable eye diseases such as glaucoma, thereby enhancing the breadth and depth of primary care services.

## Strengths and limitations of the study

A key strength of this study was its comprehensive training model, which emphasised progressive skill acquisition, competency-based assessment, and participant feedback. The structured design of the training ensured that CHWs were not only exposed to theoretical knowledge but also given ample opportunities to practise and refine their skills. The inclusion of pre- and post-tests allowed for objective measurement of knowledge gains, while the practical evaluation assessed operational competence. Together, these methods provided a robust assessment of the feasibility of training CHWs to use portable devices for glaucoma detection.

The study also demonstrated the practicality of deploying multiple portable devices simultaneously in routine primary care settings. This holistic approach reflects real-world service delivery more accurately than studies focusing on a single device. Moreover, the overwhelmingly positive feedback from CHWs suggests that training in eye health is both acceptable and engaging for this cadre of workers.

However, the study has limitations. The 3 day training period may not have been enough for participants to fully master device use and troubleshooting, and long term skill retention was not assessed. The small sample and purposive selection limit generalisability to other settings. The training did not include simple tests such as the oblique flashlight test or Van Herick’s technique for detecting angle closure glaucoma, and it did not cover the identification and referral of children with congenital or infantile onset glaucoma. The lack of a standardised national curriculum for CHW training in eye health also poses challenges for sustainable scale up.

### Future research

Future research should aim to validate these findings in larger, more diverse populations of CHWs across different regions of Nigeria and sub-Saharan Africa. Longitudinal studies are needed to assess skill retention over time and the impact of periodic refresher training on maintaining competence. Comparative studies involving ophthalmic assistants or optometrists could provide benchmarks for evaluating CHW performance in glaucoma detection.

Additionally, research should examine the accuracy of CHW referrals compared with specialist diagnoses, as well as the cost-effectiveness of integrating portable device use into primary health care. Policymakers will require evidence on the financial and logistical feasibility of scaling up such interventions nationally. Developing a standardised, accredited curriculum for training CHWs in eye health would also help ensure consistency, quality, and sustainability across programmes.

Beyond glaucoma, portable eye health devices could be used to detect other conditions such as cataract, diabetic retinopathy, and uncorrected refractive errors, further expanding the role of CHWs in community-based eye care. Embedding eye health more broadly into CHW responsibilities could contribute significantly to achieving universal health coverage and the goals of the WHO WRV.

## Conclusion

Portable devices have the potential to significantly revolutionise eye care service delivery, especially in LMICs, due to their relatively low cost, ease of operation, and low maintenance requirements. The ability to train CHWs to effectively use these devices is a critical step toward addressing the challenge of accessibility and affordability in the prevention and early detection of blinding diseases such as glaucoma. In regions where specialised eye care is scarce or inaccessible, these portable devices provide an opportunity for primary healthcare providers to detect glaucoma at an early stage, refer patients for further treatment, and reduce the burden of preventable blindness. Training CHWs to use these devices could ultimately contribute to better eye health outcomes, alleviate the strain on specialised ophthalmic services, and improve the quality of life for individuals in underserved communities.

## Data Availability

The original contributions presented in the study are included in the article/supplementary material, further inquiries can be directed to the corresponding author.
